# Cornu Ammonis Regions–Antecedents of Cortical Layers?

**DOI:** 10.3389/fnana.2017.00083

**Published:** 2017-09-26

**Authors:** Audrey Mercer, Alex M. Thomson

**Affiliations:** Department of Pharmacology, School of Pharmacy, University College London, London, United Kingdom

**Keywords:** neocortex, hippocampus, pyramidal cells, interneurones, development, neuronal circuitry, neocortical columns

## Abstract

Studying neocortex and hippocampus in parallel, we are struck by the similarities. All three to four layered allocortices and the six layered mammalian neocortex arise in the pallium. All receive and integrate multiple cortical and subcortical inputs, provide multiple outputs and include an array of neuronal classes. During development, each cell positions itself to sample appropriate local and distant inputs and to innervate appropriate targets. Simpler cortices had already solved the need to transform multiple coincident inputs into serviceable outputs before neocortex appeared in mammals. Why then do phylogenetically more recent cortices need multiple pyramidal cell layers? A simple answer is that more neurones can compute more complex functions. The dentate gyrus and hippocampal CA regions—which might be seen as hippocampal antecedents of neocortical layers—lie side by side, albeit around a tight bend. Were the millions of cells of rat neocortex arranged in like fashion, the surface area of the CA pyramidal cell layers would be some 40 times larger. Even if evolution had managed to fold this immense sheet into the space available, the distances between neurones that needed to be synaptically connected would be huge and to maintain the speed of information transfer, massive, myelinated fiber tracts would be needed. How much more practical to stack the “cells that fire and wire together” into narrow columns, while retaining the mechanisms underlying the extraordinary precision with which circuits form. This demonstrably efficient arrangement presents us with challenges, however, not the least being to categorize the baffling array of neuronal subtypes in each of five “pyramidal layers.” If we imagine the puzzle posed by this bewildering jumble of apical dendrites, basal dendrites and axons, from many different pyramidal and interneuronal classes, that is encountered by a late-arriving interneurone insinuating itself into a functional circuit, we can perhaps begin to understand why definitive classification, covering every aspect of each neurone's structure and function, is such a challenge. Here, we summarize and compare the development of these two cortices, the properties of their neurones, the circuits they form and the ordered, unidirectional flow of information from one hippocampal region, or one neocortical layer, to another.

On his deathbed in 1934, Santiago Ramón y Cajal wrote to his last student, Rafael Lorente de Nó, continuing a life-long discussion: “the mouse is not a good choice for the study of cortical circuits because of its paucity of short-axon cells…”^*^.

^*^Ramón y Cajal S. Letter to Lorente. 1934. Courtesy of Dr Francisco Alvarez, translation by Rafael Yuste. Lorente de Nó, like many since, did not agree.

## Principal cells

### Origins of principal cells in the neocortex

This section draws heavily upon many excellent reviews (Nadarajah and Parnavelas, 2002; López-Bendito and Molnár, 2003; Cheung et al., 2007; Molnár et al., 2012; Tabata et al., 2012; Evsyukova et al., 2013; Tan and Shi, 2013; Sekine et al., 2014; Hoerder-Suabedissen and Molnár, 2015; Kawauchi, 2015; Molnár and Hoerder-Suabedissen, 2016). (Montiel et al., 2016, Figure 1; https://www.ncbi.nlm.nih.gov/pmc/articles/PMC4832283/figure/cne23871-fig-0001/).

Principal cells, i.e., glutamatergic, spiny excitatory pyramidal and spiny stellate cells are generated in the ventricular zone (VZ) from asymmetrical division of progenitor radial glial cells (Miyata et al., 2001; Noctor et al., 2001) or basal progenitors in the subventricular zone (Noctor et al., 2004; Shitamukai et al., 2011; Wang et al., 2011). Post-mitotic neurones then move to the multipolar cell accumulation zone (MAZ) just above VZ. There they stay (1–3 days: Kitazawa et al., 2014), extending and retracting multiple fine processes (Tabata and Nakajima, 2003; Tabata et al., 2009), until they begin to move toward the intermediate zone (IZ) below the cortical plate (CP, future gray matter). In IZ, the neurones become bipolar and “climb” through the CP toward the marginal zone (MZ), using the process of a single radial glial cell as a scaffold (Rakic, 1972; http://rakiclab.med.yale.edu/research/; Kawauchi, 2015 Figures 1, 2, https://www.ncbi.nlm.nih.gov/pmc/articles/PMC4595654/figure/F1/; https://www.ncbi.nlm.nih.gov/pmc/articles/PMC4595654/figure/F2/). Their leading process becomes anchored in MZ, they part company with their radial glial partners and their somata are pulled up to lie beneath MZ, or CP (Nadarajah et al., 2001; Sekine et al., 2011; Kitazawa et al., 2014).

The earliest born pyramidal cells form the deepest layer, L6. As later born neurones migrate, they pass through L6, forming sequentially more superficial layers. Phylogenetically, development of an additional germinal layer, the subventricular zone (SVZ) coincides with the appearance of L2-4 and emergence of the mammalian six layered neocortex (Noctor et al., 2004; Wu et al., 2005); the layers of phylogenetically older, three layered cortices being considered equivalent to L1, L5, and L6. The primate goes further, adding an additional germinal layer, the outer subventricular zone (OSVZ) (Lukaszewicz et al., 2005), which in the Macaque results in correspondingly deeper supragranular layers (Hoerder-Suabedissen and Molnár, 2015; Montiel et al., 2016). (Molnár et al., 2006, Figures 5, 7, https://www.ncbi.nlm.nih.gov/pmc/articles/PMC1931431/figure/F5/; https://www.ncbi.nlm.nih.gov/pmc/articles/PMC1931431/figure/F7/).

### Origins of principal cells in the hippocampus

Hippocampal CA regions are often considered to contain a single pyramidal cell layer, though developing CA regions also include neurones generated in SVZ (Kitazawa et al., 2014). Whether this population remains distinct from those arising in VZ is unclear. Likewise, whether there is an ordered, birth-date-dependent, inside-out layering of *stratum pyramidale* in hippocampal CA regions appears a matter for debate. However, while there may not be the wide range of pyramidal classes to be found in neocortex, CA1 pyramids are not all identical; to quote Lorente de Nò (1934) “*There are two types of pyramids, superficial and deep ones. The superficial are arranged in one or two very dense rows. The deep pyramids are grouped into several less dense rows below*.…”'

“Deep” refers to the earliest born cells whose migration terminates close to the germinal layers, adjacent to the ventricles, cells destined to lie adjacent to *stratum oriens* (Supplementary Figure 1. http://uclsop.net/interneuron-reconstruction/ca1-pyramid). Superficial pyramids, lying adjacent to the future *stratum radiatum*, are born 1–2 days later, contain the calcium binding protein, Calbindin (Cb), Zinc (Slomianka and Geneser, 1997) and reelin and are more commonly dye-coupled, one with another (indicative of electrical gap junctions) than pyramids devoid of Cb (Baimbridge et al., 1991; Mercer et al., 2006; Mercer, 2012 for review). They also express different transcription factors (similar to deep/superficial expression in neocortex: Britanova et al., 2005; Dobreva et al., 2006; Leone et al., 2008); the deep cells expressing Sox5 and the superficial cells, SatB2 (Slomianka et al., 2011) and Zbtb20 (Xie et al., 2010), which may control Cb-expression (Nielsen et al., 2010). Even in CA regions disrupted by mutations, like Reeler, pyramids maintain separate identities, forming distinct—if mislocated—layers. Later born cells spend longer in MAZ; regions of IZ devoid of cell bodies, become filled with axons after early born cells have passed through (Kitazawa et al., 2014) and connections with these axons may delay migration of later born multipolar neurones (Altman and Bayer, 1990b). Later born superficial pyramids fire earlier, with higher probability during sharp wave ripples, while deep pyramids more frequently exhibit place fields, fields that are more plastic. Deep pyramidal firing correlates more with specific landmarks, superficial with general context (Geiller et al., 2017, for review).

The CA3 hippocampal plate (HP, future *stratum pyramidale*) becomes apparent at E18 (rat), expanding to adopt its pronounced curved profile by E22 (Altman and Bayer, 1990b). This expansion presents long migration paths for neurones generated in VZ, especially those destined to lie near the dentate gyrus. Radial movement from the tangential migratory stream into developing CA3 *stratum pyramidale* is promoted by Math2 (transcription factor), while continued tangential migration toward the developing dentate gyrus is promoted by Prox-1 (Sugiyama et al., 2014). CA3 pyramids are—on average—born earlier than CA1 neurones (E16-E20); with those that will lie close to CA1 born first (Bayer, 1980; Altman and Bayer, 1990b,a). Like CA1 pyramids, newly generated CA3 pyramidal cells move from VZ to MAZ, becoming multipolar and waiting there longer than CA1 cells (Nakahira and Yuasa, 2005); possibly for innervation from dentate gyrus (Altman and Bayer, 1990b). That neurones born at the same time in dentate gyrus and CA regions, exhibit similar gene expression patterns and become preferentially connected with each other (Deguchi et al., 2011), has important implications for functional circuitry.

A distinct CA2 region, delineated by PCP4 immunostaining, is thought to emerge postnatally and to reach adult dimensions at P21 (San Antonio et al., 2014). Until relatively recently, rodent hippocampi were thought not to contain a CA2 region and further developmental detail has yet to materialize.

### Sister cells and local connectivity

Future neocortical pyramids climb radially, up a single, straight, radial glial process to reach their final destination. Sister cells, resulting from divisions of a single progenitor, therefore come to lie in a narrow, radially oriented “column.” “The Radial Unit Hypothesis,” proposed by Rakic (1988) as the anatomical basis for neocortical columnar architecture (Mountcastle, 1957), states that the position of a neurone's precursor in VZ determines its final horizontal coordinates, while its birth date determines its radial position.

In contrast, sister CA pyramids become distributed horizontally, often across large areas of *stratum pyramidale* (Kitazawa et al., 2014; Sugiyama et al., 2014). The leading processes of radial glial cells that direct migration here are not always straight, or radially oriented, as in neocortex. In CA1, they often curve, to run almost parallel with layer boundaries (Nakahira and Yuasa, 2005). In addition, migrating multipolar neurones continue to extend and retract processes in HP, contacting several radial glial cells, selecting one and migrating along a different path, in a “zig-zag” manner (Nowakowski and Rakic, 1979; Kitazawa et al., 2014; Xu et al., 2014; Hayashi et al., 2015, for review).

This raises an interesting question about local pyramidal interconnectivity. Neocortical pyramidal cells preferentially innervate their sisters (Yu et al., 2009; Costa and Hedin-Pereira, 2010), which exhibit, for example, similar orientation preferences in primary visual cortex, V1 (Li et al., 2012). Electrical coupling may precede sister-to-sister chemical synapse-formation since this similarity in orientation preference is lost when gap junctions are blocked from P1-7, or Cx26 (connexin 26) mutated (Li et al., 2012). If similar orientation preferences do not result solely from another influence, such as preferential innervation of sister-cells by common afferent axons, the physical separation of sister neurones may be a significant factor in determining whether they “wire together.”

In both mature neocortex and CA regions, powerful electrical synapses form between closely neighboring pyramids (CA1, Baimbridge et al., 1991; CA1-3, neocortex, Mercer et al., 2006; Mercer, 2012); an average of 25% of steady state and 10% peak action potential (AP) voltage change transferring to the coupled cell. The resultant post-junctional “spikelets” can trigger overshooting APs. Quite unlike electrical junctions between interneurones (neocortex: Gibson et al., 1999; Tamás et al., 2000; Amitai et al., 2002; Simon et al., 2005; hippocampus: Fukuda and Kosaka, 2000; Meyer et al., 2002; Allen et al., 2011), these junctions form between somata and proximal apical dendrites; hence the very high electrical-coupling ratios. Vertically distributed neocortical sister-cells are, therefore, well positioned for such connections; horizontally distributed sister-CA pyramids are not (unless axon-axonic electrical junctions are also involved: Schmitz et al., 2001; Wang et al., 2010). However, other factors, such as the preferential innervation of CA1 pyramids by CA3 pyramids exhibiting similar gene expression patterns (Deguchi et al., 2011), may also contribute to the emergence of functionally related sister-cell groups across regions. Indeed, CA1 sister pyramids rarely develop electrical or chemical synapses with each other, but they do receive common input from nearby fast-spiking (FS, but not non-FS) interneurones and exhibit synchronous synaptic activity, indicative of common excitatory drive (Xu et al., 2014). During development, early born GABAergic “hub” neurones with long range connections (which later develop into projection interneurones: Picardo et al., 2011) facilitate such connectivity (Bonifazi et al., 2009; Villette et al., 2016). Spiny cells connected by chemical synapses receive common excitatory (Song et al., 2005; Yoshimura and Callaway, 2005; Kampa et al., 2006) and inhibitory inputs (Xu et al., 2014) and deliver coincident outputs, more frequently than unconnected cells, and input-convergence from electrically coupled pyramids via chemical synapses is high (5:11, Bannister and Thomson, 2007).

### Development of the wide range of neocortical pyramidal cell classes

(Table 1; Cheung et al., 2007; Hoerder-Suabedissen and Molnár, 2012, 2013, 2015; Hayashi et al., 2015, for reviews).

**Table 1:** Summary of properties of pyramidal cells in cortical layers 3–6, for references, see text.

**Layer 6 spiny cell type**	**Structural features**		**Excitatory inputs**	**Outputs**	**Firing characteristics**
	**Dendrites**	**Axons**			
L6 Cortico-thalamic pyramidal cells	Small-medium, upright pyramidal cells. Apical dendritic tuft in L4	Axon ascending to L4 (some also to lower L3). Drumstick-like branches in L4	Reciprocal from specific thalamic nuclei. From L6 cortico-cortical pyramids	To specific thalamic nuclei and nRT. Local outputs predominantly to L6-L4 GABAergic interneurones (synapses on shafts of aspiny dendrites). Facilitating EPSPs to all targets	Modest Accommodation and Adaptation. Almost tonic discharge in response to maintained depolarization.
L6 Cortico-thalamic pyramidal cells	Short, small-medium, upright pyramidal cells. Apical dendritic tuft in L5	Ascending to L5. Some with drumstick-like branches	Thalamus. L6 cortico-cortical pyramids	To specific and non-specific thalamus and local L5/6 interneurones. Facilitating EPSPs to all targets	Modest Accommodation and Adaptation. Almost tonic discharge in response to maintained depolarization.
L6 Cortico-cortical pyramidal cells (latexin positive)	Small-medium “pyramids.”Dendrites confined to L5/6. Several structural classes: short upright pyramids, bipolar, inverted and multipolar “pyramids”	Long horizontal branches confined to L5/6	Other local and distant cortical neurones	Preferentially innervate cortical pyramids with depressing EPSPs	Rapidly and powerfully adapting. Spike inactivation can be “rescued” with ramp-shaped current
L6 Claustrum-projecting pyramidal cells	Tall, upright, long thin apiical dendrite to L1-no tuft	Long horizontal branches confined to L5/6	Other local and distant cortical neurones	Claustrum, L5/6 pyramids with depressing EPSPs	Near tonic firing
**Layer 5 spiny cell type**	**Structural features**		**Excitatory inputs**	**Outputs**	**Electrophysiology**
	**Dendrites**	**Axons**			
L5 Large burst-firing pyramids, upper L5	Thick basal dendrites L5, Thick apical with tuft L3-L1	Largely confined to deep layers, short branches	Local inputs include other large and small L5 cells and a powerful focused input from deep L3 as well as distant cortical and subcortical. Most inputs accocunted for	To non-specific thalamic nuclei, superior colliculus, pons, spinal cord (targets depending on cortical region). Depressing EPSPs to most targets	Intrinsic burst-firing superimposed on a depolarizing envelope. Resting Potential *in vitro* near firing threshold.
L5 smaller cortico-thalamic pyramids	Smaller upright pyramids. Slender apical dendrites terminating in L2/3 with little/no tuft	Ascending to L2/3 and horizontal branches	No reciprocal input from thalamus	Large boutons to non-specific thalamus. Depressing EPSPs	Adapting and accommodating firing pattern
L5 smaller cortico-cortical pyramids, incl. transcallosally projecting cells	Smaller upright pyramids. Slender apical dendrites terminating in L2/3 with little/no tuft	Long horizontally oriented	Other cortical pyramidal cells, local and distant	Local and distant cortical neurones with largely depressing EPSPs	Radidly adapting and accommodating firing pattern
**Layer 4 spiny cell type**	**Structural features**		**Excitatory inputs**	**Outputs**	**Electrophysiology**
	**Dendrites**	**Axons**			
L4 pyramidal cells predominantly innervating L4 cells	Often small, simple cells. A modest number of slender dendrites, basals in L4, apical obliques in L3, with a tuft in L1	Local axonal arbor and a descending arbor with sparse branching in L5 and/or L6	From local L4 cells (28% of input), 6% from specific thalamus (large, potent *en-passant* boutons on dendritic shafts), 45% from L6 corticothalamic (small boutons, on spines). Almost none from L3. Remainder currently unaccounted for	Predominantly other L4 cells. Strength and probability falling off rapidly with separation. Proximal, basal dendritic inputs. Brief, depressing EPSPs	Rapidly adapting and accommodating
L4 pyramidal cells preferentially innervating L3 cells	Often small, simple cells. A modest number of slender dendrites, basals in L4, apical obliques in L3, with a tuft in L1	Strong, ascending, topographically precise input to L3 and descending projection with sparse branching in L5 and/or L6		Predominantly to L3 cells. Pyramids more than interneurones. Proximal, basal dendritic inputs. Brief, depressing EPSPs	Brief, short interspike interval spike train followed by brief afterdepolartization, slow hyperpolarization then tonic firing
L4 Spiny stellate cells	Often small, simple cells, with slender dendrites largely confined to L4. No apical dendrite	Ascending topographically precise input to L3, descending projection with sparse branching in L5			Probably similar to the above
**Layer 3 spiny cell type**	**Structural features**		**Excitatory inputs**	**Outputs**	**Electrophysiology**
	**Dendrites**	**Axons**			
L3 pyramidal cells	Well developed basal and apical oblique dendrites and a tuft in L1. Largest cells close to L4 border	Dense, fairly narrow ramifications in L3 and L5, not in L4 (but see text for mouse)	Inputs from L4 and thalamus to deep L3 proximal basal dendrites. Tall, brief, depressing EPSPs. High hit-rate inputs from other local L3 pyramids. Cortical and thalamic inputs account for most synapses. 97% of L3 pyramid-pyramid inputs onto spines of less proximal basal and apical oblique dendrites	Dense local innervation of L3 pyramids and interneurones and patchy, long distance terminal axonal arbors. Dense, very high probability innervation of large (not small) L5 pyramids sharing the same vertical axis. To interneurones in L4 that have dendrites in L3, but not to spiny L4 cells. Transcallosal projections	Very negative resting potentials −80mV *(in vitro*). “Typical” adapting/accommodating pyramidal cells

*With the exception of presynaptic L6 cortico-thalamic pyramids, all pyramidal inputs to FS, parvalbumin-immunopositive interneurones recorded were depressing and all excitatory inputs to SOM cells were facilitating*.

*Few studies in L4 have systematically correlated anatomy with electrophysiology and connectivity. Some characteristics, like their inputs and the descending projections may, or may not be common to 2 or more subclasses*.

Both the inside out, sequential formation of L2-L6 and the sequential generation of the different classes of pyramids destined for a single layer, ensure a shifting environment as new cells are born and begin to migrate. Distinct expression patterns of a large array of genes coding for transcription factors and regulators, growth factors, receptors, peptidase inhibitors, acetylation regulatory factors, glycoproteins, kinases, guidance-, signal-, adhesion-, and extracellular matrix- molecules, reelin, its receptors and their downstream signaling pathways, not to mention those genes for which no function has yet been found, have been identified in sub-populations of progenitors and differentiating neurones. The milieu into which a neurone is born, those it travels through as it migrates from VZ/SVZ, through MAZ, IZ and into CP/HP and where it eventually establishes itself, are both temporally and spatially regulated. One example is the postmitotic expression of Sox5 in subcortically projecting deep layer pyramids and Satb2 in corticocortically projecting, superficial layer cells (Slomianka et al., 2011). The latter, if induced to express Sox5 ectopically, lose their corticocortical projections and instead project subcortically (Arlotta et al., 2005; Alcamo et al., 2008; Britanova et al., 2008; Fishell and Hanashima, 2008, for review).

#### Neocortical layer 6 pyramidal cells

Like other layers, L6 contains several distinct classes of spiny, glutamatergic principal cells (Thomson, 2010 for review). The birth-dates of two broad groups are distinguished by their expression of latexin (carboxypeptidase-A inhibitor). Corticortical cells, which express latexin are born after corticothalamic cells which do not: E15 *cf* E14 (Arimatsu and Ishida, 2002). (Thomson and Lamy, 2007, Figure 5, https://www.ncbi.nlm.nih.gov/pmc/articles/PMC2518047/figure/F5/).

Only earlier born, corticothalamic pyramids receive direct thalamic input. One subclass of these upright cells, with apical dendritic tufts in L4, send narrow, ascending axonal arbors to L4 (and sometimes lower L3) where it terminates with characteristic short, drumstick-like side branches. These neurones project subcortically to “specific,” or primary sensory thalamic nuclei and to *nucleus reticularis thalami* (nRT, the thalamic inhibitory nucleus) (Zhang and Deschenes, 1998). All L6 corticothalamic pyramids fire with minimally accommodating/adapting, near tonic discharge and preferentially innervate GABAergic cells with consistently facilitating patterns of transmitter release (West et al., 2006). In neocortex, >90% of their synaptic boutons contact dendritic shafts of non-spiny neurones (White and Keller, 1987), including L4 (Tarczy-Hornoch et al., 1999; Beierlein et al., 2003) and L5 interneurones (Staiger et al., 1996). Despite their frequent innervation of parvalbumin (PV) interneurones (which receive depressing inputs from all other pyramidal classes), L6 corticothalamic pyramids elicited facilitating EPSPs (excitatory postsynaptic potentials) in *all* cell types studied, including ventroposterior, posterior medial thalamic and nRT neurones. This contrasts with the depressing EPSPs elicited by L5 pyramids in posterior medial thalamic nucleus (Reichova and Sherman, 2004).

The second corticothalamic subclass, more commonly found in deep L6, projects to *both* specific and non-specific thalamic regions such as PO (posterior thalamic group) The apical dendrites of these short, upright pyramids and their ascending axons typically terminate in upper L5. Neither subgroup of corticothalamic cells has long horizontal axon collaterals in the infragranular layers, all branches turn toward the pial surface.

In cats and primates, where L4 subdivisions are morphologically and functionally distinct, subclasses of corticothalamic cells are found, each with its apical dendritic branches and axonal ramifications restricted to a specific L4 sublayer (Lund, 1987; Wiser and Callaway, 1996). Further specificity was demonstrated by a study combining *in vivo* physiology and morphology in cat V1 (Hirsch et al., 1998b,a). L6 pyramidal simple cells (“simple” implying significant direct input from lateral geniculate nucleus, LGN and resembling “specific” thalamocortical pyramids) targeted L6 and/or L4, layers rich in simple cells. L6 complex cells (receiving integrated, rather than “specific” corticothalamic inputs), targeted L2/3 and L5, layers rich in complex cells.

In striking contrast to corticothalamic pyramidal cells, are the rapidly adapting, corticocortical L6 pyramids, which preferentially innervate other pyramidal cells with “depressing” synapses and display an array of morphologies (Mercer et al., 2005), short, upright pyramids whose apical dendrites terminate in L5, bipolar cells and inverted pyramids. All have long, horizontal axons confined to L5/6 (Zhang and Deschenes, 1998; Mercer et al., 2005), some crossing areal boundaries. Their pronounced spike accommodation/adaptation cannot be overcome by injecting larger square-wave current pulses; these only result in more rapid and profound soma/initial segment Na^+^ channel inactivation. However, a ramp-shaped current superimposed on the original threshold square-wave pulse, activates tonic firing of overshooting APs, probably originating at more distant axonal locations and propagated, or reflected passively, back to the soma (unpublished; Stuart et al., 1997; Colbert and Pan, 2002; Clark et al., 2005, for axonal spike-initiation).

The near tonically firing claustrum-projecting cells form the third major L6 pyramidal class, with long slender apical dendrites that reach L1 without forming a tuft there and a broad, axonal arbor confined to L5 and L6 (Katz, 1987). Like L6 corticocortical cells, claustrum-projecting pyramids preferentially innervate pyramids locally, with “depressing” synapses (Mercer et al., 2005).

L6 is often perceived as a predominantly thalamo-recipient layer, but only corticothalamic pyramids receive powerful, direct thalamic input. Nor do corticocortical cells receive powerful excitation from neighboring thalamo-recipient corticothalamic cells. Some descending inhibitory projections from L4 ramify in L6, but excitatory projections from superficial layers are often narrow and sparse. Binzegger et al. (2004, cat V1) estimated the numbers of synapses supplied to each layer by cortical and LGN relay neurones. When compared with estimates based on stereological analysis (Beaulieu and Colonnier, 1985), the estimates for excitatory synapses were within 10% for L2/3 and L5, but differed by 32% for L4 and 70% for L6. Many additional corticocortical, or subcortical inputs are required to account for the boutons in these thalamo-recipient layers. (Thomson, 2010, Figure 4; https://www.ncbi.nlm.nih.gov/pmc/articles/PMC2885865/figure/F4/).

In cats and primates most subplate (SP) neurones disappear during development; a few remaining in the underlying white matter as interstitial neurones (Kostovic and Rakic, 1980; Luskin and Shatz, 1985; Valverde et al., 1989; Naegele et al., 1991). However, in rodents, degeneration in SP is less dramatic and the SP becomes L6b (or L7) (Valverde et al., 1989; Ferrer et al., 1992).

#### Neocortical layer 5 pyramidal cells

The principal inputs to L5 (and to L5 pyramidal dendrites in L3) are local and more distant corticocortical projections. In turn, large L5 pyramidal cells project to many subcortical targets, including “non-specific” thalamic nuclei, superior colliculus, pons and spinal cord (targets depending on cortical region). Smaller L5 pyramids project to other cortical and subcortical regions and transcallosally, to contralateral neocortex.

Upper L5 contains the largest neocortical pyramids (only these approaching the size and spine densities of CA pyramids). In cat V1, the large cells that project to the colliculi, and/or the pons (Hallman et al., 1988), have thick apical dendrites with well-developed apical tufts in L1/2 and substantial basal dendritic arbors largely contained within L5. The largest, Betz cells (Betz, 1874), are found in motor cortex and project via the corticospinal tract to the spinal cord. Large L5 cells display a stereotypical “intrinsically burst-firing” behavior (Connors et al., 1982); the burst of two or more, high frequency spikes being superimposed on a well-developed depolarizing envelope, due to activation of a dendritic Ca^2+^ spike (Purpura and Shofer, 1965; Llinas, 1975; Larkum et al., 1999). The short interspike-interval train of two or more spikes, typical of rapidly adapting/accommodating neurones (smaller L5-, L6 corticocortical-, and some L4 pyramids), should not be confused with stereotypical bursts (though it often is); it is not superimposed upon, or triggered by a stereotypical depolarizing envelope and does not occur repetitively if the cell is held near spike threshold. Quasi burst-firing can also be elicited in adapting cells electrically coupled to intrinsic bursters (Mercer et al., 2006). The local axons of large L5 pyramids arborize almost exclusively within the deep layers while the smaller pyramids also project to the superficial layers (Larsen and Callaway, 2006).

A significant input to these large, intrinsically burst-firing pyramids comes from smaller L5 pyramids. Whether these smaller pyramids project to non-specific thalamus, or to other cortical regions was not determined. In adult rat L5, small adapting pyramids were 10 times more likely to innervate large burst-firing pyramids than vice versa (unpublished data: Thomson and West, 1993; Deuchars et al., 1994). Large L5 pyramidal cells that are close neighbors are, however, relatively densely interconnected (hit rate of 1:10: Markram, 1997). Large, but not small L5 pyramids, also receive a dense, highly focussed, input from deep L3 pyramids (hit rate > 1:4, Hübener et al., 1990; Thomson and Bannister, 1998, for reconstructions of cat L5 pyramids).

The apical dendrites of small-medium corticothalamic and corticocortical L5 pyramids are slender, rarely extend beyond L2/3 and have no significant apical tuft. L5 corticothalamic pyramids provide large boutons to non-specific thalamic regions from which they receive no reciprocal inputs, in contrast to L6 corticothalamic cells which are reciprocally connected with “specific” thalamic nuclei and deliver small boutons (Van Horn and Sherman, 2004). A separate population of smaller, shorter L5 pyramids projects transcallosally (Hübener et al., 1990; Kasper et al., 1994). Transcallosal cells are found in all layers except L1 (Kasper et al., 1994).

#### Neocortical layer 4 spiny cells: pyramidal cells and spiny stellate cells

If we can assume that three layered cortices in some non-mammalian species do a perfectly good job, as far as the requirements of those animals are concerned, receiving e.g., sensory information in one layer (equivalent to mammalian L6) and integrating that information with signals from elsewhere, in the adjacent layer (equivalent to L5), which then sends instructions to other brain regions, we could ask why a presumed need for more complex and sophisticated organization and integration of that information could not have been achieved simply by expanding these two existing layers. Whether this was attempted in some long lost evolutionary dead-end, we may never know, we can only assume that such an attempt did not survive. Instead, a new germinal zone, SVC and three new layers (L2-L4) to which the SVC contributes spiny cells, were added. Interestingly, these new layers repeat the pattern established in deeper layers: peripheral input into L4, with integration within, and distribution from L2/3.

Layer 4 contains two broad classes of spiny excitatory cells. Typically, the basal dendrites of L4 pyramidal cells are contained within L4 with apical oblique dendrites in L2/3 (though they receive little or no input from local L3 pyramids) and an apical dendrite extending into L1, often forming a small tuft there. Spiny stellate cells lack an apical dendrite, most or all of their dendrites are confined to L4 (Lund, 1973). Perhaps the most striking distinguishing feature of L4 spiny neurones in rat and cat, especially when compared with the “chunkier” pyramids in adjacent layers, is their simple (Rojo et al., 2016) and delicate appearance (Bannister and Thomson, 2007).

Despite being a major thalamo-recipient layer, thalamocortical inputs to L4 contribute only 6% of the synapses onto spiny stellate neurones in cat V1 and up to 22.9% in mouse (Benshalom and White, 1986), terminating predominantly on dendritic spines via large *en-passant* boutons. In contrast, ascending L6 corticothalamic pyramidal axons, form synapses with small boutons, but provide 45% of the excitatory inputs onto L4 spiny cells (cat, primate, Lund et al., 1988, for review). In primate V1 axons from area MT terminate in L1, L4B, and L6. This contrasts with other so called “feedback” connections from “higher” visual areas terminating in L1; projections that might more meaningfully be termed “cognitive” or “attentional” feed-forward. In V2 they terminate primarily in L1 and L5 or L6 (Rockland and Knutson, 2000). A further 28% of the excitatory input to L4 spiny cells originates from within L4 (cat, Ahmed et al., 1994). Despite the small numbers of thalamocortical inputs, their large boutons provide secure, faithful transmission of early presynaptic spikes, albeit followed by pronounced presynaptically mediated depression. Thalamocortical synapses have three times more release sites than those of local circuit axons, with higher release probabilities, making the average thalamocortical connection several times more effective (Gil et al., 1999, mouse S1), *at the start* of a spike train.

The axons of L4 spiny neurones make dense, topographically precise ascending projections to L3 and sparse descending projections to upper L5 (rat, cat) (Valverde, 1976; Parnavelas et al., 1977; Feldman and Peters, 1978; Gilbert, 1983; Burkhalter, 1989) where they innervate pyramids and (less commonly) interneurones (Thomson et al., 2002). Pyramids and spiny stellates contribute to these projections and both provide relatively narrow, horizontal arbors within L4. In cat V1, some spiny cell axons make most of their synapses within L4, others form a larger proportion in L3 (Binzegger et al., 2004), a finding supported by morphometric analysis coupled with paired recordings in rat barrel cortex (Lübke et al., 2003) and one that correlates with distinct electrophysiological classes (below). The sparse projection, from L4 to L6, appears to originate predominantly with pyramids (unpublished).

The firing patterns of adult L4 pyramidal and/or spiny stellate cells correlated with distinct connectivity patterns. Stereotypical intrinsic bursts were rare. Around 60% displayed rapid spike accommodation and frequency adaptation (recoverable with a superimposed ramp) and innervated other L4 spiny cells. The remaining 40% produced a short train of 3–5 short interspike interval spikes, followed by a brief afterdepolarization, then a slow afterhyperpolarization upon which a spike-train of increasing interspike interval was superimposed. These cells preferentially innervated L3 pyramids (Bannister and Thomson, 2007). This raises interesting questions about patterns of synaptic input *in vivo* and how they might interact with the cells' inherent firing characteristics; tonic input to L3, phasic to neighboring L4 pyramids in reponse to maintained depolarization.

Connectivity ratios for pairs of L4 pyramids were relatively low (1:18 adult rat; 1:14 cat V1), with no selection for firing characteristics, and fell off extremely rapidly with increasing horizontal somatic separation. All identified synaptic contacts onto spiny cells (L4 and L3, rat and cat) were onto proximal primary, secondary, and tertiary, electrotonically compact basal dendrites, and all EPSPs were brief and depressing (Bannister and Thomson, 2007).

#### Neocortical layer 3 pyramidal cells

Layer 3 pyramidal cells are “typical” pyramids, with adapting firing patterns, well developed basal and apical oblique dendrites and an apical dendrite forming a tuft in L1. The largest are close to the L4 border. More superficial L2 cells are, naturally, very short with almost no apical dendrite. L3 pyramidal axons ramify densely in L2/3 delivering depressing inputs to other L3 pyramids with a hit rate of 1:3 that falls off only gradually with distance. Their main descending axons typically pass through L4 without branching to ramify in L5, in rat (Lorente de Nó, 1922; Burkhalter, 1989), cat (O'Leary, 1941; Gilbert and Wiesel, 1983; Kisvárday et al., 1986), and primate (Spatz et al., 1970; Lund et al., 1993; Yoshioka et al., 1994; Kritzer and Goldman-Rakic, 1995; Fujita and Fujita, 1996), where they innervate large L5 pyramids;. Somewhat surprisingly some deep L3 pyramids in mice have substantial axonal arbors within L4 (Larsen and Callaway, 2006). Their targets in L4 are of interest, because although L3 pyramids innervate L4 interneurones that have dendrites projecting into L3, they rarely, if ever excite L4 spiny cells in adult rat or cat (Bannister and Thomson, 2007).

An additional, distinctive firing pattern has been described in cat V1—chattering cells (Gray and McCormick, 1996). These neurones generate extremely fast intrinsic spike-bursts, with an intraburst firing rate up to 800 s^−1^ and a repeat rate of 20–70 s^−1^, in response to visual stimuli or suprathreshold current injection. During visual stimulation these cells exhibit pronounced oscillations in membrane potential that are largely absent at rest. All chattering cells recovered after dye-filling were typical L2/3 pyramidal neurones.

L3 receives a substantial trans-callosal input, larger than that to L5 (Porter and White, 1986) and pyramids in both layers project trans-callosally, often to topographically related cortical areas. In L2/3 of rat barrel cortex, 97% of the connections, both local and distant, made by L3 pyramidal axons are onto dendritic spines. This is a striking target preference (seen in all layers), when only 80% of all asymmetrical synapses in L3 are onto spines (White and Czeiger, 1991) and L6 corticothalamic cells preferentially innervate aspiny dendritic shafts in L4 and L6 (Elhanany and White, 1990). In primate visual, motor and somatosensory cortex, L3 (and to a lesser extent L5) cells also provide dense innervation of patches of cortex a few 100 μ wide and up to a few millimeters from the injection site within L1–3 (Levitt et al., 1993, 1994). In prefrontal cortex, a narrow stripe-like, rather than a patchy pattern is apparent (Levitt et al., 1993).

Deep L3 pyramids can also receive thalamocortical inputs from primary sensory thalamus, again, largely to proximal basal dendrites, though the further they are from the L4 border, the weaker this input becomes (White and Hersch, 1982). A major part of the projection from the pulvinar (the most caudal thalamic group, with roles in attention and oculomotor behavior) also terminates in L3 extrastriate visual areas (Rockland et al., 1999). The dense, focussed, excitatory input from L4 spiny cells onto L3 pyramids also terminates proximally, on first, second, or third order basal dendrites (Thomson et al., 2002; Thomson and Bannister, 2003), while the many inputs from other L3 pyramids are located more distally (mean 97 μm *cf*. 69 μm: Feldmeyer et al., 2002), on both basal and apical oblique dendritic branches. Proximal basal synapses result in taller, narrower EPSPs (excitatory postsynaptic potentials) than more distal inputs.

#### The relationship between dendritic location and EPSP size and shape

This relationship is partly due to the smoothing of current transfer over the length of a cable with resistance and capacitance (Rall, 1962) and partly to the activation of voltage-gated ion channels distributed with unique patterns of surface expression across somata, axons and dendrites of each class of neurone (Nusser, 2009, 2012, for reviews). Amongst the conductances whose density increases with distance from the soma, perhaps the most studied has been the rapidly inactivating K^+^ current, I_A_. The I_A_ α-subunit Kv4.3 clusters in neocortical pyramidal dendrites and dendritic spines (Burkhalter et al., 2006) and I_A_ density increases as I_Na_ decreases more distally in large L5 pyramidal basal dendrites (Kampa et al., 2006). In CA1 pyramidal dendrites, the density of Kv4.2 also increases along the soma-dendritic axis (Kerti et al., 2012), although the gradient was shallower than expected from dendritic recordings of I_A_ (e.g., Hoffman et al., 1997; Sun et al., 2011; Nestor and Hoffman, 2012); a discrepancy that might result from involvement of other I_A_ α-subunits, auxiliary subunits, or modulators of channel conductance.

In mouse L3 pyramids, selective blockade of Kv4.2/4.3 enhanced glu-EPSPs activated by focal glutamate-uncaging at single spines. It also promoted activation of fast, dendritic spikes by summed glu-EPSPs at proximal dendritic locations and of slower, all-or-none, stereotypical, depolarizing events at proximal-intermediate dendritic locations (A Biro, A Bremaud, A. Ruiz, unpublished). Without channel-blockers these additional events required near simultaneous activation at 7–8 closely neighboring locations. Such events would enhance responses of L3 cells to thalamocortical inputs. However, two excitatory synapses provided by any one presynaptic axon rarely, if ever innervate the same pyramidal dendrite, let alone 7 or 8. They distribute across the dendritic tree on different branches, albeit at similar electrotonic distances from the soma. How frequently 7–8 presynaptic terminals, each from 7 to 8 different presynaptic neurones, all impinging on a single dendritic compartment, are activated simultaneously in life, is difficult to predict. More distal inputs e.g., from other L3 neurones or cortical regions may lower the threshold for such events (Branco and Häusser, 2011), perhaps when attention to a behaviourally important input is required. In both mouse A1 and V1 L4, local circuit activation amplified and prolonged thalamocortical responses, without altering frequency or direction selectivity and with spectral range and tuning (auditory), or with frequency and direction selectivity (visual) preserved (Li et al., 2013a,b).

### Unidirectional flow of excitation in neocortex and hippocampus

Both cellular and circuit properties appear to have developed to preserve the integrity of the signals arriving from the periphery. In L4, thalamocortical input arrives in proximal postsynaptic compartments that are near optimal for rapid, faithful transmission to soma/axon. The signals carrying this information may then be enhanced or suppressed in L3 and additional features, like direction in V1, computed there. However, the purity of salient feature representation in the direct thalamocortical signal is not compromised by excitation from other layers; from cells dealing with more highly integrated and processed information. The flow of excitatory input from the thalamus is unidirectional: from thalamus (and L6) to L4, L4 to L3, and L3 to L5 and transmitted thence to other cortical and subcortical regions. The strength of a response may be altered by coincident inputs from the recipient layer, from other layers, or regions; the response may be tuned, or suppressed by inhibition in L4 activated from elsewhere, but its fundamental integrity is preserved (Thomson et al., 2002; Thomson and Lamy, 2007, for review).

**Figure 1:**
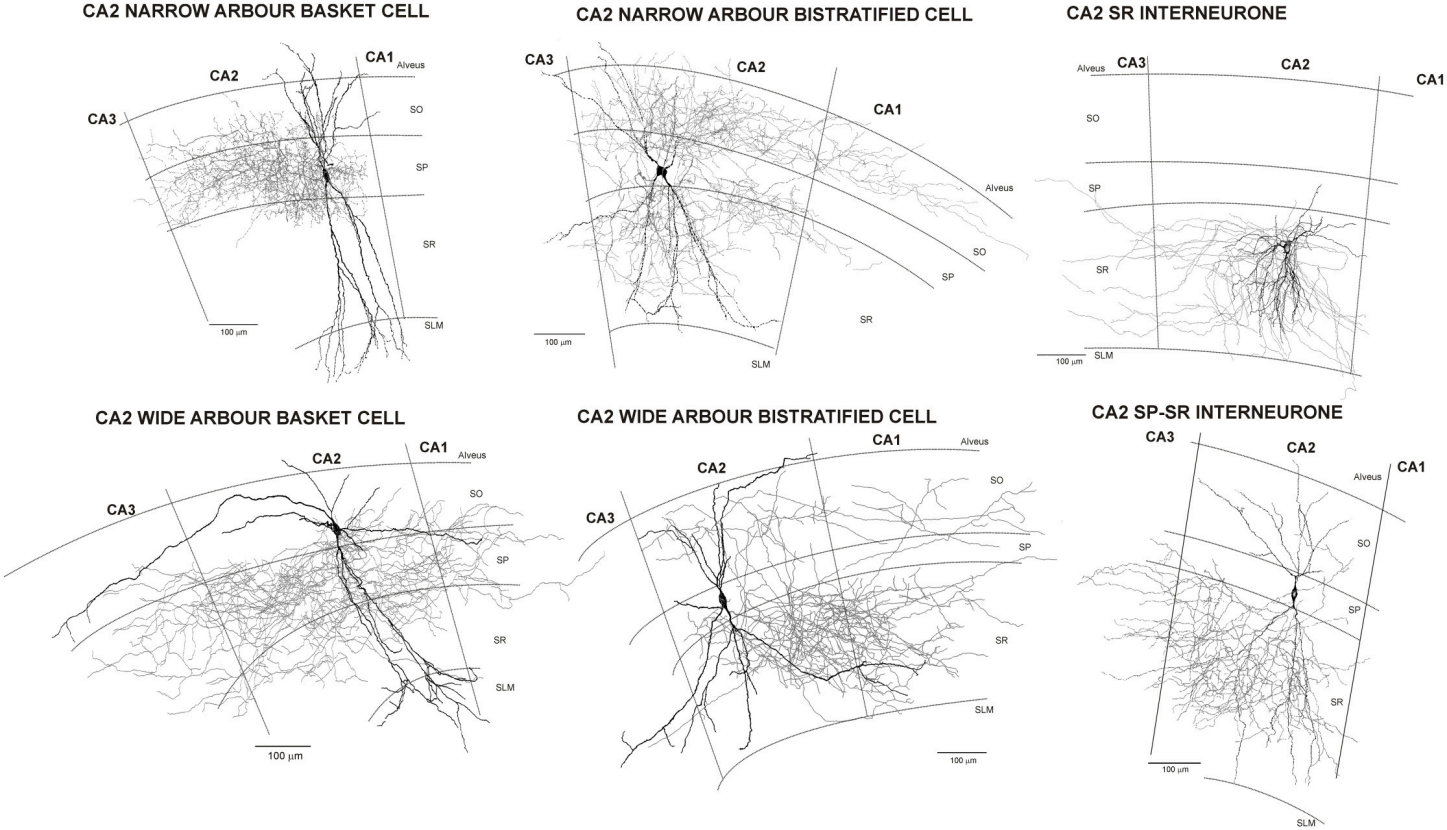
Reconstructions of CA2 interneurones filled during intracellular recordings in adult rat hippocampus (from Mercer et al., 2007, 2012). The largest population of interneurones recorded and filled in CA2 were basket cells. Like those in CA1, CA2 basket cells had dendrites that extended through *stratum oriens*, sometimes entering the alveus, and through *stratum radiatum* and into *stratum lacunosum moleculare*. Their axons arbourized extensively in *stratum pyramidale* and in some, also in very proximal *stratum oriens* and/or *radiatum* (wide axonal arbor basket cells). Two distinct subtypes of CA2 basket cells were identified. The first (CA2 narrow dendritic arbor basket cells) resembled those of CA1 with a narrow, aspiny dendritic arbor and axon confined to CA2. In contrast, both the axons and dendrites of the CA2 wide dendritic arbor basket cells, extended into all three CA-subfields and the horizontally oriented, distal dendritic branches were sparsely spiny. Similarly, two subtypes of CA2 bistratified cells were reported, CA2 narrow and wide dendritic arbor bistratified cells. The dendrites of both subtypes extended through *stratum oriens* and *radiatum* without entering *stratum lacunosum moleculare*, those of wide dendritic arbor cells extending further horizontally than is typical of CA1 bistratified cells and becoming sparsely spiny. Bistratified cell axons ramified in both CA2 and proximal CA1, but stopped abruptly at the CA2/CA3 border. The somata of CA2 SP-SR interneurones were found in *stratum pyramidale* and their dendrites extended to *stratum oriens*, branched extensively in *stratum radiatum*, rarely penetrated SLM, but often extended horizontally to CA1 and CA3. Their axons emerged from the soma and arbourized almost exclusively in *stratum radiatum* of CA2. The axons and dendrites of CA2 stratum radiatum, Reelin-immunopositive interneurones ramified predominantly in CA2 *stratum radiatum*, with a few axonal branches extending into neighboring regions.

In this, the neocortical circuit is strongly reminiscent of hippocampus where dentate granule cells, activated by inputs from enthorinal cortex, send excitatory inputs to CA3, CA3 pyramids send excitatory inputs to CA2 and CA1 and CA1 pyramids project to different layers of the enthorinal cortex via the subiculum. CA1 pyramids do not project “back” to excite CA3 pyramidal cells. The CA1 neurones that innervate CA3 and dentate gyrus are not glutamatergic pyramidal cells, but GABAergic interneurones—“back projection cells” (below and Supplementary Figure 1, http://uclsop.net/interneuron-reconstruction/backprojection/). Some CA2 pyramids, as well as interneurones, do project “back” to CA3 (Figure 1; Mercer et al., 2007, 2012; Mercer, 2012), but their targets there have yet to be identified.

One of the reasons such an elegant organization in neocortex has been difficult to accept, or even imagine (e.g., Binzegger et al., 2004) is the apparent chaos that results from neocortical layering, *cf* the discreet regional organization in hippocampus. In neocortical layers 2–6, there are somata, axons and apical and basal dendrites arising from many different classes of neurones whose somata reside in any of these layers. The inputs from other layers, from other cortical and subcortical areas may terminate neatly in specific layers, or sublayers, but what do they find there but a jumbled multiplicity of potential targets. To propose that these axons can seek out and connect only to specific targets amongst this confusion—not only to connect to certain subclasses of neurones, but to specific postsynaptic compartments belonging to those neurones—seemed quite preposterous.

It is, however, the case. Those of us not skilled in the art may view electron micrographs of the neocortical neuropil with a sense of horrified bewilderment, but axons and dendrites apparently know with whom they are destined to communicate and make it their business to find each other. We have come to accept that GABAergic interneuronal axons can find and innnervate very specific targets, eschewing all others in their path, so why have we assumed that excitatory axons make synaptic contacts indiscriminately, with any old neuronal element they happen to pass? (see also Markram et al., 2015). Different pyramidal classes are born on different embryonic days, express different combinations of gene products at different times during migration and differentiation, migrate through gradually changing chemical and physical environments, halt for different lengths of time *en route* and receive different incoming synapses. Neocortical pyramidal (unlike interneuronal) axons may often follow almost linear, class-specific trajectories, but their targets are more flexible—employing spines to sample the environment and twisting and bending to capture an attractive input.

We do not yet know which molecules are involved in this synaptic partner-identification; they are likely to be different at each class of synapse (defined by the subclasses of pre- and post-synaptic neurones). But we do know that each class of synapse, so defined, displays its own unique characteristics: specificity in transmitter(s) used, pre- and post-synaptic receptors inserted, frequency-dependent patterns of transmitter release, postsynaptic compartments involved and thereby the modulation of each input by cable and voltage-gated properties and by other nearby inputs.

### Subplate neurones and afferent axons

#### Neocortex

There is considerable evidence that epigenetic cues are required for the final differentiation of neocortical neurones, still somewhat multi-potent on arrival. Obvious candidates for such cues are in-growing axons, particularly, perhaps, thalamocortical axons in primary sensory regions (López-Bendito and Molnár, 2003, for review).

Connections between the neocortex and subcortical structures course through the internal capsule, a thick fiber tract lying between the caudate nucleus and thalamus. Subplate neurones, diverse in site of origin, birth date, survival and gene expression, exhibit a range of morphologies and axonal projection patterns (Hoerder-Suabedissen and Molnár, 2013), including pioneer axons to the emerging internal capsule and commissural fibers of the early hippocampus (Sarnat and Flores-Sarnat, 2002). The subplate zone becomes a “waiting compartment” in which thalamocortical-, basal forebrain cholinergic-, callosal, commissural, and ipsilateral corticocortical-afferents cease growing until an appropriate environment or signal emerges.

Early born GABAergic cells migrate tangentially from their germinal zone in the LGE (lateral ganglionic eminence) and into the MGE (medial ganglionic eminence), forming a stream of cells between the MGE and globus pallidus (E11.5 to E14). These “corridor” cells form a permissive pathway through which thalamocortical axons can grow (López-Bendito et al., 2006) (Molnár et al., 2012: Figures 1 and 2; https://www.ncbi.nlm.nih.gov/pmc/articles/PMC4370206/figure/F1/; https://www.ncbi.nlm.nih.gov/pmc/articles/PMC5040712/figure/F2/). Otherwise chemical repellents and the structure of the PSPB (pallial-subpallial boundaries): high cell-density, and a radial glial fascicle running across the trajectory of thalamocortical axons, would hinder their onward growth toward the cortex. Cortigofugal axons may also assist the forward growth of thalamocortical axons through this barrier (Molnár et al., 1998; Molnár and Butler, 2002).

Like many cortical neurones, most thalamic neurones are born between E13 and E19 (rat), the LGN, for example, between E12 and E14. By E16/E17, nuclear differentiation in thalamus has begun and both neocortex and dorsal (specific) thalamus have started to generate prospective reciprocal connections. To reach their destinations, these axons must overcome and cross several emerging barriers, or boundary zones: the diencephalic-telencephalic (DTB) and (PSPB) form transient barriers to axon growth, but interestingly, also a route for early born migrating neurones that form the permissive corridor. A largely transient population of pioneering subplate neurones sends the first projections to the internal capsule (IC) and beyond; though the axons of other cortical neurones actually invade and innervate specific thalamic nuclei first. However, without subplate projections, thalamocortical axons cannot traverse the PSPB to enter the telencephalon. Moreover, subplate ablation at this time, prevents formation of ocular dominance columns, inhibition in L4 does not mature, barrels are disrupted and spindle activity abolished (Hoerder-Suabedissen and Molnár, 2015).

By P0, axons from L6 have reached the ventrobasal thalamic nucleus (primary somatosensory) and over the next 4 days they invade and form a barreloid pattern. Corticothalamic fibers do not, however, ramify within LGN until the eyes open and spontaneous activity begins. By E16-19 thalamocortical axons have accumulated in the subplate, but they also wait, extending horizontal collaterals that may facilitate reorganization of maps at a later date, until peripheral afferents innervate the appropriate dorsal thalamic nucleus (Molnár et al., 2012).

Here we see an important change in the forward growth of thalamocortical axons from the external route seen in lower vertebrates lacking a six layered cortex, where they run over the developing cortex, to the internal route of mammals, via the corpus callosum. The midline repellent, Slit2, redirects the migration of corridor neurones, switching thalamic axons from an external to a mammalian-specific internal path (Bielle et al., 2011). It is proposed that this switch allowed the neocortex to grow radially. Interestingly, the hippocampus is deep in the brain and bounded by dense fiber tracts. Perhaps it was not able to grow in this way.

Having accumulated in the subplate, the growth of thalamocortical axons into neocortex is prevented if SNARE-complex proteins, essential for AP-driven, Ca^2+^-dependent transmitter release (though not spontaneous, “miniatures,” Ramirez and Kavalali, 2011) are knocked out. With the arrival of thalamocortical axons, transient circuits form between thalamic axons, subplate and L4 neurones. Multiple interactions now control the growth of- and connections made by- incoming axons and the development of cortical neurones and circuits. For example, two extracellular molecules: NRN1 (Neuritin-1, a GPI-anchored neuronal protein that modulates neurite outgrowth) and VGF (a nerve growth factor), both manufactured by thalamic cells and transported to their neocortical terminals, promote L4 spiny stellate dendritic growth—selectively (Sato et al., 2012). Lhx2 promotes activity-dependent L4 dendritic growth toward thalamic afferents, by inducing the transcription factor BBtbd3 (Wang et al., 2017), while several neurotrophins are implicated in the critical stages during which precise thalamocortical connections are made (Ma et al., 2002; Yamamoto and Hanamura, 2005). As L4 and its thalamic inputs mature postnatally, spiny stellate cells receive a transient input from SOM interneurones in L5b, which themselves receive thalamic input. Development of thalamic input to spiny stellates is delayed in the absence of this transient input (Marques-Smith et al., 2016), while thalamic afferents are misdirected to inappropriate barrels when Proteoglycan-2 (PRG-1, a phospholipid- interacting molecule) is knocked out (Cheng et al., 2016).

#### Hippocampus

As in neocortex, expression patterns demonstrate that pyramidal classes are predestined at E15.5 while they are still in IZ. For example, SCIP (POU domain transcription factor), is present in future CA1 pyramids (Frantz et al., 1994; Tole et al., 1997), while KA1 (GluR subunit) is expressed in future CA3 neurones (Wisden and Seeburg, 1993; Tole et al., 1997) and many regulators that control neurogenesis in neocortex also act here (Urbán and Guillemot, 2014).

From LII and LIII of the entorhinal cortex information from many subcortical structures is relayed to the hippocampus via the perforant path, providing powerful input to the molecular layer of the dentate gyrus and to distal apical dendritic tufts of CA1-3 pyramidal cells in *stratum lacunosum moleculare*. Mossy fibers project from dentate granule cells to CA3 *stratum lucidum*, innervating the most proximal apical dendrites of CA3 pyramids with huge boutons. In turn, CA3 pyramidal axons (Schaffer collaterals) project to *stratum radiatum* and *oriens* of CA1. The hippocampus also sends information to and receives inputs from subcortical regions: medial septum, cingulate gyrus, mammillary bodies, thalamus and amygdala as well as regions of association cortex.

The precise position of CA2 in this unidirectional trisynaptic pathway has been unveiled more recently (Chevaleyre and Piskorowski, 2016; Dudek et al., 2016; for reviews). LII of the entorhinal cortex provides strong, proximal excitation to CA2 pyramidal cells via dentate and mossy fiber synapses in *stratum lucidum* (Kohara et al., 2014). CA2 is also thought to receive direct input from LIII of the entorhinal cortex in *stratum radiatum* and *lacunosum moleculare* (Chevaleyre and Siegelbaum, 2010) in addition to Schaffer collaterals (Chevaleyre and Siegelbaum, 2010; Jones and McHugh, 2011). In turn, CA2 pyramids project preferentially to calbindin-negative, deep CA1 pyramids which lie adjacent to *stratum oriens* (Kohara et al., 2014). CA2 pyramids also project “back” to the supramammillary nucleus (Tamamaki et al., 1988; Cui et al., 2013) and in some cases, back to LII of the medial enthorinal cortex (Rowland et al., 2013).

During development, a projection from CA1 non-pyramidal cells to the medial septum, (hippocampo-septal pathway) (Supèr and Soriano, 1994) develops before the reverse, septo-hippocampal projection: E15 vs. E17 (mouse) (for parallel studies in rat and involvement of Cajal-Retzius cells: Ceranik et al., 1999, 2000). Chemo-repulsive semiphorins repel septal axons, promote growth cone collapse and may contribute to target selection; GABAergic septo-hippocampal fibers terminate preferentially on sema3C-expressing GABAergic interneurones, while cholinergic septo-hippocampal fibers terminate on sema3E- and sema3A-expressing CA pyramidal and dentate granule cells (Pascual et al., 2005).

By E17, LIII entorhinal axons are ramifying densely and exclusively in *stratum lacunosum moleculare*. Invasion of the dentate comes later, but by E19, the first entorhinal axons begin to ramify there, predominantly in the outer molecular layer (Supèr and Soriano, 1994). Commissural fibers first enter the contralateral hippocampus at E18 and arborize in *stratum radiatum* and *oriens*, along with the Schaffer collaterals. The earliest commissural fibers to enter the dentate gyrus are seen even later, at P2, terminating in the inner zone of the molecular layer and the hilus. Thus, as in neocortex, incoming pathways do not meander indiscriminately; they invade their ultimate target layers and regions from their earliest appearance, some following paths marked by early born, non-pyramidal neurones.

Again, a host of genes selectively expressed at different times, in different locations and in different cell classes in the developing hippocampus appear to contribute to its normal development. For examples, see Fazzari et al. (2010) for signaling with Nrg1(Neuregulin1, a ligand for ERBB3 and 4) and ErbB4 (receptor tyrosine kinase, an epidermal growth factor receptor); Silva et al. (2015), for LGI1 (Leucine-rich, glioma inactivated 1) in both neocortex and hippocampus; (Mingorance et al., 2004), for tempero-spatial patterns of Nogo expression and its debated involvement in perforant path development (Urbán and Guillemot, 2014).

#### A note on Cajal-Retzius cells (Cajal, 1891, 1899a,b, 1904; Retzius, 1893)

Large numbers of calretinin-expressing (CR), bipolar or multipolar Cajal-Retzius neurones appear in the molecular layer of the developing CP, becoming distributed through all layers. Collaterals of their thick primary axon make synaptic contact first with pyramidal cells in emerging L6, then sequentially with pyramids in L5 to L2 (del Rio et al., 1995). In hippocampus they become densely innervated by afferent axons from entorhinal cortex, whose ramification in CA *stratum lacunosum moleculare* and dentate outer molecular layer is severely reduced if Cajal-Retzius cells are ablated. Up to 90% of these cells disappear during development; the remainder form a sparse population in adult neocortical L1 (del Rio et al., 1995), hippocampal *stratum lacunosum moleculare* and the dentate gyrus outer molecular layer (Del Río et al., 1997). Cajal-Retzius neurones produce GABA, possibly ACh, calmodulin, PV(parvalbumin) and CR and neuropeptides. They express important mediators of radial neuroblast migration and lamination of the cortical plate: Reelin (a secreted extracellular matrix protein, essential for the normal “inside-out” development of neocortical layering), Lis1 (a motor protein Dynein-regulator), and Dscam (Down syndrome cell adhesion molecule). In addition to forming the first intrinsic synaptic circuits of the cortical plate and its first afferent and efferent connections with subcortical structures, Cajal-Retzius neurones may contribute to ocular dominance column-formation, to regulation of neurogenesis, and to cortical repair (Sarnat and Flores-Sarnat, 2002, for review).

## GABAergic interneurones

### Origins of the many classes of GABAergic cortical interneurones

(Meyer and Wahle, 1988; Wonders and Anderson, 2006; Batista-Brito and Fishell, 2009; Vitalis and Rossier, 2011; Miyoshi et al., 2013; Li and Pleasure, 2014; Wamsley and Fishell, 2017, for reviews; Yavorska and Wehr, 2016, Figure 1, https://www.ncbi.nlm.nih.gov/pmc/articles/PMC5040712/figure/F1/; Batista-Brito and Fishell, 2009, Figure 3, https://www.ncbi.nlm.nih.gov/pmc/articles/PMC4465088/figure/F3/; Cauli et al., 2014, Figure 1, https://www.ncbi.nlm.nih.gov/pmc/articles/PMC4067953/figure/F1/; Jovanovic and Thomson, 2011, Figure 1, https://www.ncbi.nlm.nih.gov/pmc/articles/PMC3139172/figure/F1/; Brandão and Romcy-Pereira, 2015, Figure 1, https://www.ncbi.nlm.nih.gov/pmc/articles/PMC4412069/figure/F1/).

In humans, 65% of neocortical interneurones develop from Mash1-expressing progenitor cells of the VZ and SVC. Mash1 is a gene responsible for differentiation of GABAergic neurones and is also expressed in the subpallium (Letinic et al., 2002; Jakovcevski et al., 2011). In most mammals, however, the majority of GABAergic cortical interneurones are born in the subpallium, divisible into lateral (LGE), medial (MGE), and caudal (CGE) ganglionic eminences and preoptic area (POA). Interneurones expressing PV are born in ventral MGE (vMGE); those expressing SOM in dorsal MGE (dMGE); interneurones expressing the 5HT3 receptor (5HT3R, ionotropic serotonin receptor, Lee et al., 2010) plus cells variously expressing CR, CCK, VIP, SOM, PV, reelin and NPY (neuropeptide Y) are born in CGE (Lee et al., 2010). Finally, a mixed population of CR, CCK, VIP (vasoactive intestinal polypeptide), SOM, PV, reelin, and NPY cells are born in POA. Between E9.5 and E15.5, PV cells in vMGE, SOM cells in the dMGE and cells expressing reelin, SOM, CR, are born. 5HT3R cells are born later (E12.5–E15.5). The orphan nuclear receptor COUP-TFII is expressed in the CGE and in hippocampal interneurone-specific interneurones. It is required, with COUP-TFI, for caudal migration of cortical interneurones (Cauli et al., 2014), while activation of 5HT3AR promotes migration and appropriate positioning of CGE-derived reelin-cells (Murthy et al., 2014) (Yavorska and Wehr, Figure 2, https://www.ncbi.nlm.nih.gov/pmc/articles/PMC5040712/figure/F2/).

The interneurones then migrate tangentially toward the cortex. Corticofugal axons expressing TAG-1 (an axonal glycoprotein) provide a pathway for early-born MGE interneurones, while later-born interneurones migrate preferentially along axons lacking TAG-1 (McManus et al., 2004; Denaxa et al., 2011). Along two main migratory streams (in MZ and SVZ) they interact with soluble chemo-attractants and-repellents. For example, Cxcl12, interacting with its receptors, Cxcr4, Cxcr7, is a potent chemo-attractant for MGE-derived interneurones and required for normal positioning of these interneurones (Li et al., 2008; López-Bendito et al., 2008). Activation of GluRs and GABA_*B*_Rs, promotes tangential migration of interneurones into the cortex (Luhmann et al., 2015). Early born, SOMinterneurones, in receipt of strong thalamic input at this time, innervate PV interneurones and pyramids. These transient circuits promote maturation of thalamocortical input to PV interneurones (Tuncdemir et al., 2016; see above, for the influence of transient circuits involving thalamorecipient-SOM interneurones, on spiny stellate maturation). CGE-derived interneurones must insinuate themselves into the cortex even later, after many other interneurones are in place. To migrate properly and develop appropriate processes, they need network activity and, after P3, glutamate-release (De Marco García et al., 2011).

Each subpallial region expresses different combinations of transcription factors and both birth-date and -location influence the classes of interneurones generated. By P0, a large part of their fate has been defined by their own genetic programmes, but most interneurones arrive after pyramidal neurones and early interneurones have populated the cortex. Additional factors fine tune their structure and function: interactions with pyramidal cells influence their final positions, electrical activity regulates late acquisition of neurochemical identity and of soluble factors, which also influence chemical identity and thereby the relative proportions of interneuronal subtypes (Brandão and Romcy-Pereira, 2015, for review).

#### Ambiguity and uncertainty in the classification of interneurones

Many recent studies have used rodents—young enough for many neuronal properties still to be maturing. Neonatal voltage gated channels, transporters and receptors are replaced during the first few postnatal weeks, resulting in a dramatic—up to four fold—reduction in the time course of many electrophysiological events. This “juvenile” period is also a time of synapse proliferation and pruning, and the speed and complexity of short term synaptic dynamics (Thomson, 2000a,b, 2003) increase in parallel. Some of the ambiguity encountered in attempts to classify cortical interneurones could result from cells at different stages of maturity; a day or two at these ages could make quite a difference: Kvα1 (Butler et al., 1998); SK2 (Cingolani et al., 2002); Kv3.2 (Tansey et al., 2002); Kv3.1b (Du et al., 1996); speeding of AMPA-R-EPSPs, P8 *cf* P35; shortening of synaptically released glutamate waveform, P8-P18 (Cathala et al., 2003, 2005); GABA_*A*_-R α6-subunit expression, P7 *cf* P30 (Tia et al., 1996; time course of NMDA-R mediated EPSCs (Hestrin, 1992; Cathala et al., 2000); NMDA-R subunits (Farrant et al., 1994); switch from FLIP to FLOP GluR splice variants P8-14 (Monyer et al., 1991). (Batista-Brito and Fishell, 2009, Figure 5, https://www.ncbi.nlm.nih.gov/pmc/articles/PMC4465088/figure/F5/).

Moreover, reconstructions of “juvenile” cells typically demonstrate rather limited axonal ramification.

Studying a more restricted developmental stage might, therefore result in a “tidier” picture. However, cortical interneurones have to establish their own territories within a field of already established cortical layers, sublayers and regions; environments, moreover, that continue to change throughout development and in ways not entirely prescribed genetically. Following detailed studies of the crab stomatogastric ganglion, Marder and Prinz (2002) concluded that “…*similar neuronal and network outputs can be produced by a number of different combinations of ion channels and synapse strengths. This suggests that individual neurons of the same class may each have found an acceptable solution to a genetically determined pattern of activity, and that networks of neurons in different animals may produce similar output patterns by somewhat variable underlying mechanisms*….” It is perhaps not surprising, therefore, that while many properties are common to all members, where a given clearly definable subclass exists, others may be subject to variation and modification by the existing environment. It is only necessary to study the convoluted trajectories of interneuronal axons (and pyramidal dendritic branches) to appreciate how thoroughly they explore their environment for appropriate synaptic partners.

### Hippocampal interneurones

(Klausberger et al., 2003, for review) (Figure 1. Supplementary Figure 1 for 3D reconstructions of CA1 interneurones). (Klausberger and Somogyi, 2008, Figures 1,2, https://www.ncbi.nlm.nih.gov/pmc/articles/PMC4487503/figure/F1/; https://www.ncbi.nlm.nih.gov/pmc/articles/PMC4487503/figure/F2/; Bezaire and Soltesz, 2013, Figure 1, https://www.ncbi.nlm.nih.gov/pmc/articles/PMC3775914/figure/F1/; Markram et al., 2004).

Hippocampal interneurones are generated in much the same way and in the same regions as neocortical interneurones, though they take a more caudal path to their destination. They must also become integrated into an existing network, but the organization of that network, with only one principal cell layer and major pathways spatially separated, is more straightforward.

#### Proximally targeting hippocampal interneurones

Two broad classes of interneurones target somata/proximal dendrites and axon initial segments of pyramidal cells, respectively. Many of their axonal branches become significantly—if sporadically—myelinated and their synaptic boutons are large and contain mitochondria; facilitating the fast, precisely timed, proximal inhibition they provide.

**Basket cells** “Baskets” of axons bearing large synaptic boutons that surrround principal cell somata were first described in cerebellum (Golgi, 1883, 1906) then elsewhere (Cajal, 1888, 1906; Lorente de Nó, 1922; Kritzer and Goldman-Rakic, 1995; Buhl et al., 1997; Ali et al., 1998; Tamás et al., 1998). A hippocampal interneurone destined to inhibit pyramidal somata has little choice but to innervate *stratum pyramidale*. Similarly, to sample all excitatory inputs controlling activity in its target cells, it extends its dendrites across all layers, from *stratum oriens* to *lacunosum moleculare*—an easily identifiable, classical CA basket cell. Some basket cell axons can also extend to proximal *stratum oriens* and *stratum radiatum* (wide arbor basket cells, Supplementary Figure 1, http://uclsop.net/interneuron-reconstruction/basket).

Three types of CA1 basket cells, the majority otherwise fairly similar in their appearance, are distinguished by immunoreactivity for PV, CCK/VIP; or CCK/VGLUT3 (Katona et al., 1999; Somogyi et al., 2004) and CB1R (type-1 cannabinoid receptor: Takács et al., 2015) (Pawelzik et al., 2002, for distributions of CA1 PV and CCK interneurones). CA1 CCK basket cells receive less synaptic input than PV baskets, with proportionally more inhibition, suggesting that they do indeed subserve different rôles (Mátyás et al., 2004) and unlike PV basket cells, whose cell bodies lie predominantly in *stratum pyramidale*, CCK basket somata are also found in *stratum oriens* and *radiatum*, i.e. their sampling of incoming information also has a different bias.

For neurones with such a similar overall structure and specific target preference, it is surprising perhaps that PV and CCK basket cells originate in different subpallial regions: PV interneurones in vMGE, CCK interneurones in POA or CGE and may be born later. PV interneurones (devoid of other common markers) are typically fast spiking (FS) and deliver fast IPSPs mediated by α1β2/3γ2-GABA_A_Rs to pyramids, while many CCK basket cells display adapting firing patterns, have broader action potentials (Pawelzik et al., 2002) and activate α2β2/3γ2-GABA_A_Rs on pyramids. The different pharmacologies of these receptors (hippocampus: Pawelzik et al., 1999, 2003; Thomson et al., 2000; neocortex: Ali and Thomson, 2008) and the behavioral effects of manipulating their efficacy (Möhler et al., 2002) suggest that PV baskets mediate pharmacological sedation and contribute to anti-convulsant therapies, CCK basket cells (and possibly axo-axonic cells, Nusser et al., 1996) promote anxiolysis (Möhler et al., 2002), while certain dendrite-preferring interneurones, acting on α5β1γ2-GABA_A_Rs (Pawelzik et al., 1999, 2003; Ali and Thomson, 2008) influence cognition (Rudolph and Möhler, 2014).

**Chandelier, or Axo-axonic cells** innervate pyramidal axon initial segments in deep *stratum pyramidale* and proximal *oriens*. Their cartridge bouton arrays are only partially coincident with basket cell axonal arbors (Buhl et al., 1994b). For chandeliers with somata in *stratum pyramidale* this and the often distinctive claw-like appearance of their apical dendritic terminal branches as they extend into *stratum lacunosum moleculare* assist their identification (Pawelzik et al., 2002) (Supplementary Figure 1, http://uclsop.net/interneuron-reconstruction/axo-axonic). For *stratum oriens* axo-axonic cells with horizontal dendrites, see Ganter et al. (2004).

#### Dendrite-targeting interneurones

At least nine classes of CA1 interneurones preferentially innervate pyramidal dendrites. Their termination zones suggest that each class selectively innervates dendritic regions also receiving a particular afferent pathway, or combination thereof. The names they have acquired often reflect this preference (Klausberger et al., 2003; Klausberger and Somogyi, 2008; Bezaire and Soltesz, 2013, for reviews). As a gross generalization, dendrite-targeting interneurones have finer, unmyelinated axons and smaller, mitochondria-poor synaptic boutons than proximally targeting cells. They display a range of firing patterns, but are rarely classical FS. Those that have horizontally oriented dendrites (OLM cells being a prime example), be they in *stratum oriens, radiatum*, or *lacunosum moleculare*, in CA1 or CA2, often display an adapting firing pattern and a pronounced “sag” current in responses to large hyperpolarizing current pulses, which can elicit rebound firing and many receive facilitating EPSPs from pyramids.

**Perforant path associated** cells Perforant path associated cells whose axons and dendrites are restricted to *stratum lacunosum moleculare* respond to perforant path input by inhibiting pyramidal apical dendritic tufts that are also in receipt of perforant path input. (CCK) (Vida et al., 1998; Pawelzik et al., 2002) (Supplementary Figure 1, http://uclsop.net/interneuron-reconstruction/ppa).

**Bistratified cells** have axonal arbors ramifying in *stratum oriens* and *radiatum*, but not in *stratum pyramidale or lacunosum moleculare*. Bistratified cells with somata in *stratum pyramidale*, have dendrites that span stratum oriens and radiatum. Those with cell bodies in *stratum oriens*, have horizontal dendrites confined to *stratum oriens*.The axons of both subtypes are associated with Schaffer collateral/commissural inputs to intermediate pyramidal dendrites via α5β1γ2-GABA_*A*_Rs (Pawelzik et al., 1999; Thomson et al., 2000; Thomson and Jovanovic, 2010 for review). (Supplementary Figure 1, http://uclsop.net/interneuron-reconstruction/bistratified).

(SOM, PV, CCK) (Buhl et al., 1994a, 1996; Halasy et al., 1996; Pawelzik et al., 2002; Klausberger et al., 2004; Baude et al., 2007).

**Schaffer collateral-associated** cells innervate the same regions as bistratified cells, but receive a different combination of inputs. Their somata lie close to the *stratum radiatum-lacunosum moleculare* border and their dendrites span both these layers and *stratum oriens*. In addition to Schaffer collateral and commissural input, therefore, these interneurones receive proximal input from perforant path, but restrict their influence to the termination regions of the Schaffer/commissural inputs (CCK: Vida et al., 1998; Pawelzik et al., 2002). (Supplementary Figure 1, http://uclsop.net/interneuron-reconstruction/sca).

**Apical dendrite-innervating** cells have axonal and dendritic spans similar to those of the Schaffer collateral-associated cells, but innervate the main apical dendritic trunks of pyramids, rather than their apical oblique branches (Klausberger et al., 2005; Klausberger, 2009) (CCK).

**Oriens-lacunosum moleculare**, or **OLM** cells (Cajal, 1911; Lacaille et al., 1987; Lacaille and Williams, 1990; Buckmaster et al., 1994; Blasco-Ibáñez and Freund, 1995), have horizontal thorny dendrites restricted to *stratum oriens* (in CA1) where they receive their most powerful drive from CA1 pyramids (Blasco-Ibáñez and Freund, 1995) with facilitating EPSPs (Ali and Thomson, 1998). In CA3, OLM dendrites also project into *stratum radiatum*, where local CA3 pyramidal axons also ramify. OLM cells do not, however, innervate *stratum oriens, pyramidale*, or *radiatum*. They send one or more long axons to *stratum lacunosum moleculare*, where they form a dense arbor in the perforant path termination zone and deliver fast IPSPs, almost invisible at the soma, but apparent in distal apical dendritic recordings (Hannelore Pawelzik, 1960-2004; Hannelore Pawelzik, unpublished) (Supplementary Figure 1, http://uclsop.net/interneuron-reconstruction/olm).

(SOM: Morrison et al., 1982; Kosaka et al., 1988; Kunkel and Schwartzkroin, 1988). (mGluR1α: Ferraguti et al., 2004) (up to one third express PV weakly: Ferraguti et al., 2004; Varga et al., 2012) one (metabotropic glutamate receptor 7, mGluR7, selectively expressed in excitatory boutons contacting OLM cells: Shigemoto et al., 1996).

#### GABAergic projection neurones

(Jinno, 2009, Figure 1, https://www.ncbi.nlm.nih.gov/pmc/articles/PMC2718779/figure/F1/).

These are perhaps the group most difficult to classify and one of the smallest 4% of CA1 interneurones. Since the majority of reported cells in the following four classes have horizontally oriented dendrites confined to *stratum oriens*, it is probable that, like OLM cells, they receive strong excitatory input from CA1 pyramids and relay information about activity here to other regions. In addition to long distance projections, they have local axonal arbors in *stratum oriens* and *radiatum*.

(SOM; Jinno et al., 2007; Katona et al., 2017) PV possible: Ferraguti et al., 2004).

**Oriens-retrohippocampal projection** cells project to the subiculum. (SOM/Cb: Jinno et al., 2007; Klausberger and Somogyi, 2008). A range of subtypes project to subiculum, including an mGluR8-decorated, M2R-expressing, SOM-negative trilaminar cell (Ferraguti et al., 2005).

**Double projection** cells (Klausberger and Somogyi, 2008) project to the septum and subiculum (SOM/Cb, or CR). Some also express mGluR1α and/or NPY and up to 30% express PV weakly.

**Back-projection** cells (Sik et al., 1994; Katona et al., 2017, for *in vivo* filled cells) project to CA3 and/or dentate gyrus, sometimes crossing the fissure, which appears to be an impenetrable barrier to other neuronal processes. (PV, SOM, Cb-negative). (Supplementary Figure 1, http://uclsop.net/interneuron-reconstruction/backprojection).

**Cb-septal projection cells** project to the septum (SOM, Cb) (Gulyás et al., 2003).

**Amygdala-projecting interneurones** project from ventral CA1 *stratum oriens, pyramidale* and *radiatum*, to the amygdala (Lübkemann et al., 2015). (PV, Cb, SOM, NPY and/or CCK).

#### The neurogliaform family

The neurogliaform family (Overstreet-Wadiche and McBain, 2015, for review).

Two classes have been described, which differ predominantly in the inputs they receive and the subcellular compartments they inhibit.

**Neurogliaform** cells are often found at the *stratum radiatum-lacunosum moleculare* border, with short, fine, often highly convoluted dendrites and a dense and spatially restricted axonal arbor, positioned to inhibit distal apical dendrites of pyramidal cells; (nNOS (neuronal nitric oxide synthase), NPY, α-actinin-2, COUP-TFII) (Price et al., 2005; Fuentealba et al., 2010).

**Ivy** cells are structurally similar to neurogliaform cells, but lie close to the *stratum radiatum-pyramidale* border where they inhibit proximal pyramidal compartments. Although the GABA_A_Rs activated by ivy cells demonstrate rapid kinetics via receptors also utilized at PV basket synapses (α1β2/3γ2, unpublished results), the proximal IPSPs elicited by ivy cells are very slow. This may be due to non-synaptic, as well as synaptic release of GABA, since what appear to be synaptic vesicles in these axons are not always apposed to postsynaptic specializations (Fuentealba et al., 2008a; see also Oláh et al., 2009; Armstrong et al., 2012, for review) (nNOS, NPY). (Supplementary Figure 1, http://uclsop.net/interneuron-reconstruction/ivy).

### Interneurone-specific interneurones

(Acsády et al., 1996; Freund and Buzsaki, 1996; Freund and Gulyas, 1997) (CR and/or VIP and COUP-TFII: Gulyas et al., 1999).

**Interneurone-specific type I** somata are found in *stratum pyramidale*. Their dendrites and axons span *stratum oriens* and *radiatum*. They innervate Cb interneurones, VIP-, but not PV- basket cells and other IS-1 interneurones (CR).

**Interneurone-specific type II** somata lie near the *stratum lacunosum moleculare- radiatum* border. Their dendites run horizontally in *lacunosum moleculare*. They innervate distal *stratum radiatum*, making multiple contacts with Cb, but not PV-dendrites (VIP).

**Interneurone-specific type III** somata are found in *stratum pyramidale*, their bipolar/bitufted dendrites span *stratum oriens* through *radiatum* to *lacunosum moleculare*. Their axons innervate *stratum oriens*, where they inhibit Cb and SOM/mGluRa, interneurones including OLM cells (Acsády et al., 1996) (CR, VIP and possibly nNOS).

We cannot leave hippocampal interneurones without mentioning, however briefly, the elegant experiments in which a neurone is recorded through different e.g., states, then filled juxta-cellularly and identified; studies that demonstrate distinctive patterns of firing in relation to network rhythms such as theta and sharp wave ripples, for each class of interneurone (Klausberger et al., 2003, 2004; Fuentealba et al., 2008b, 2010; Klausberger and Somogyi, 2008; Varga et al., 2012; Katona et al., 2017).

### Can we transfer what we know about interneurones in CA regions to the neocortex?

There are around twenty, more or less distinct, classifiable classes of interneurones in CA1. Although those in CA3 remain to be explored as thoroughly, there appears to be a similar variety. In CA2, much the same profile is seen, but some unique subclass features and a *stratum pyramidale*-*stratum radiatum* interneuronal class, specific to this region, have been demonstrated (Figure 1) (Mercer et al., 2007, 2012a,c).

Some of the distinguishing features used to classify hippocampal interneurones, such as topographical relationship to specific pathways, have not been systematically applied to neocortical interneurone classification. If we look at broad classes of GABAergic neurones, those, for example that express the same markers, we find a similar picture in hippocampus and neocortex. Nearly all neocortical interneurones also belong to three broad groups, 40% expressing PV, 30% SOM and 30% 5-HT3αR, with little overlap (Rudy et al., 2011). As a broad generalization, PV interneurones (expressing neither SOM, Cb, nor CR) display fast spiking (FS) behavior, innervate proximal regions of pyramidal cells, generate fast IPSPs mediated by α1β2/3γ2 GABA_A_Rs (Ali and Thomson, 2008) and receive depressing EPSPs from pyramids (excepting L6 corticothalamic pyramids). SOM cells including bipolar and bitufted neurones, display adapting or “burst-firing” behavior, innervate pyramidal dendrites with slower IPSPs (somatic recordings) and receive facilitating EPSPs from pyramids. 5HT3R cells displaying various non-FS behaviors are often relatively small cells with small overlapping axonal and dendritic trees (Lee et al., 2010). Expression of mRNA for certain voltage-gated ion channels clusters with three major calcium binding proteins, PV, Cb, and CR, and correlates with firing characteristics: fast I_A_ K^+^ channel subunits in the PV cluster, that rapidly repolarize action potentials, reducing Na^+^-channel inactivation, would facilitate fast spiking behavior, while a T-type Ca^2+^ current in the Cb cluster that would support burst-firing behavior (Toledo-Rodriguez et al., 2004).

**Neocortical proximally targeting interneurones** include subclasses of basket and chandelier or axo-axonic cells, but with a far wider range of sizes, axonal and dendritic distributions, potential inputs and targets than in hippocampus.

**Basket cells** in neocortex have complex choices to make. Some of the pyramids that a neocortical basket cell is destined to control will receive excitatory input in several, or even in all layers, while some spiny cells, like inverted, or bipolar L6 corticocortical cells, or L4 spiny stellate cells, may receive inputs only in one. A neocortical basket cell must also choose which spiny cells it will inhibit - any or all pyramids in a given layer, or a specific subtype, perhaps one receiving only certain inputs. Some smaller basket cells have axonal arbors restricted to a single layer, or sublayer. Large basket cells often innervate more than one layer, though this choice is not indiscriminate; the axonal arbors are often restricted to two related layers, such as the two thalamorecipient layers, L4 and L6, or the integration layers, L3 and L5, with only unbranched collaterals passing through intermediate layers (e.g., L3 and L5: Lund, 1987; Lund et al., 1988; Buhl et al., 1997; L6 and L4: Lund, 1987; Lund et al., 1988; Thomson et al., 2002; Thomson and Bannister, 2003). These larger basket cells often have dendrites that extend over several layers and some, in cat and primate primary sensory regions, also generate long horizontal axonal branches that terminate in smaller, but equally dense arbors in more distant columns (Lund, 1987; Lund et al., 1988; Kritzer and Goldman-Rakic, 1995; Lund and Wu, 1997; Thomson et al., 2002; Thomson and Bannister, 2003).

Traditionally, as in hippocampus, neocortical basket cells have been found to stain either for PV, or CCK. However, neocortical basket cells have also been classified according to axonal branch length and angle, bouton frequency etc. and these parameters correlated with their potential to generate calcium binding proteins and neuropeptides (RT-PCR). “Small basket cells,” including “clutch cells” (Kisvárday et al., 1985) express mRNA for VIP and SOM or CCK and variously PV, Cb, or CR. Large basket cells express mRNA for PV or Cb and variously NPY or CCK. “Nest basket cells” express PV or Cb mRNA and approximately equal proportions mRNA for NPY, SOM, or CCK (Wang et al., 2002).

For quality reconstructions of identified neocortical basket cells and of other GABAergic interneurones: (Jones, 1975; Jones and Peters, 1984; Lund, 1987; Lund et al., 1988; Lund and Yoshioka, 1991; Lund and Wu, 1997; DeFelipe, 2002; Thomson et al., 2002; Thomson and Bannister, 2003; West et al., 2006) and for connections with these cells: (Somogyi et al., 1983; Kritzer and Goldman-Rakic, 1995; Buhl et al., 1996; Halasy et al., 1996; Tamás et al., 1997; Dantzker and Callaway, 2000; Thomson et al., 2002; Thomson and Bannister, 2003; West et al., 2006; Ali et al., 2007).

**Chandelier or Axo-axonic cells** (Inan and Anderson, 2014). Since the targets of chandelier cells are highly restricted—to pyramidal axon initial segments (Somogyi, 1977; Somogyi et al., 1982) and their function - to control pyramidal firing, is well documented (if somewhat controversial), we can assign part of their function according to the distribution of their cartridge synapses. The synapses made by some neocortical axo-axonic cells are restricted to a single layer, others to two physically separated, but related layers/sublayers. Cartridge synapses, on short, radially projecting collaterals make these cells easy to identify (Szentagothai and Arbib, 1974; Lund, 1987; Lund et al., 1988; Lund and Yoshioka, 1991; Kritzer and Goldman-Rakic, 1995; Lund and Wu, 1997; Thomson and Bannister, 2003). Although most studies identify PV as a predominant marker for chandelier cells, (DeFelipe et al., 1989; Kawaguchi and Kubota, 1997; Gonchar and Burkhalter, 1999) some primate and human L5/L6 chandeliers contain Cb (del Rio and DeFelipe, 1997) and a separate population of corticotrophin-containing cells has been described in primate, although relative proportions vary between species, layer and area (Lewis and Lund, 1990).

**Neocortical dendrite-targeting interneurones** may, like their hippocampal equivalents, sample only certain inputs and seek only those targets that receive specific inputs. We are inclined to suspect that they most probably do, in the face of little direct evidence.

**Somatostatin (SOM) dendrite-targeting interneurones** (Yavorska and Wehr, 2016), often bipolar or bitufted, have fine axons forming dense, vertically oriented arbors with small boutons, spanning one, two or more adjoining layers. They are typically adapting, or burst-firing, with broader APs than FS cells and receive facilitating inputs from pyramids (Deuchars and Thomson, 1995; Thomson et al., 1995; Thomson and Bannister, 2003), stronger inhibition from VIP interneurones than PV cells receive and deliver slower IPSPs than basket cells (somatic recordings), mediated by α5-subunit-containing-GABA_A_Rs (Ali and Thomson, 2008).

**Martinotti cells** from the dMGE, were first described as resident in L5, with a fine, dense axonal arbor extending to L1 and innervating pyramidal dendrites (Martinotti, 1889); leading some to claim, erroneously, any cell with a portion of axon drifting northwards as a “Martinotti.” They display “low threshold spiking” behavior (Kawaguchi and Kubota, 1996, 1997; Beierlein et al., 2000, 2003; Wang et al., 2004; Ma et al., 2006). This class is now agreed to include similar, but adapting/burst-firing SOM cells in superficial layers. However, L5/6 and L2/3/4 Martinotti cells do differ; two distinct populations are identifiable in GIN and X98 mice respectively (Ma et al., 2006), both populations including SOM/Cb and SOM/NPY cells and in mouse, SOM/Cb/CR or SOM/Cb/NPY (Ma et al., 2006).

Other, probably dendrite-targeting, SOM interneurones are less distinctive.

**SOM cells in L4/5 of the X94** mouse did not express Cb or NPY,
**SOM cells not labeled in X94, X98 or GIN** lines express NPY, nNOS and SPR (substance P receptor) (Xu and Callaway, 2009).

**VIP Bipolar/bitufted interneurones**, from the CGE, also containing neither PV nor SOM, often show irregular spiking behavior, supported by an I_D_-like K^+^ current (Porter et al., 1998). Their slender axonal tree preferentially innervates fine/medium caliber dendrites of other VIP cells as well as pyramids (rat: Peters, 1990; Acsády et al., 1996; Staiger et al., 1996, 1997; mouse: Prönneke et al., 2015), with boutons often closely associated with asymmetrical synapses (rat: Hajós et al., 1988). They receive high probability, depressing inputs from pyramidal cells mediated by AMPA-Rs with fast kinetics (GluR1/2 flop: Porter et al., 1998), particularly strong inputs from deep layers and stronger inputs from distant cortical areas: basal nucleus of Meynert and thalamus (Wall et al., 2016) and from PV cells (Staiger et al., 1997) than other interneurones.

**Double bouquet cells** (Cajal, 1899b; DeFelipe et al., 2006) with somata in L2/3/4 have a distinctive, narrow, axonal arbor (“horse-tail”) descending to L6, in addition to a dense local arbor (often unstained in Golgi preparations). They contain VIP or CR, commonly display a “sag” in response to hyperpolazing current and a range of firing patterns including stuttering and adapting, but not classical FS (Prönneke et al., 2015).

(rat: VIP, Kawaguchi and Kubota, 1997 or CR, primate: Lund and Lewis, 1993).

**Smaller VIP/CCK** or **VIP/CR cells** (Kawaguchi and Kubota, 1997) probably include small basket cells, like **Arcade cells**, whose axon first ascends toward the pia, then turns south, to form a cone-shaped arbor (Jones, 1975) innervating somata and proximal dendrites.

**Multipolar burst-firing** dendrite-targeting cells which are strongly interconnected (electrically and chemically) and unusually express both PV and Cb, may form an additional VIP subclass (mouse, Blatow et al., 2003).

**5HT3R cells** include subsets of later born CCK, CR and NPY expressing neurones (Lee et al., 2010; Rudy et al., 2011), from the CGE.

**Neurogliaform cells** (Cajal, 1891; Lund and Yoshioka, 1991; Lund and Wu, 1997; Armstrong et al., 2012, for review) with dense, convoluted dendritic and axonal arbors display late-spiking behavior. As in hippocampus, non-synaptic, but AP-driven, vesicular release (in addition to synaptic) may account for the slow time course of the IPSPs, the presynaptic GABAergic inhibition and activation of extrasynaptic α4βxδ-GABA_A_Rs these cells elicit (Oláh et al., 2009).

NPY (Xu and Callaway, 2009), COUP-TFII (Fuentealba et al., 2010) 5HT3aR, but not VIP (Lee et al., 2010; Rudy et al., 2011).

**COUP-TFII - Interneurone-specific interneurones?** In rat hippocampus, COUP-TFII is expressed in neurogliaform cells and basket cells in *stratum radiatum* and by CR- and/or VIP- interneurone-specific-interneurones (Fuentealba et al., 2010). This member of the steroid/thyroid-receptor family is expressed in the dMGE and CGE, in the SVZ in humans and by interneurones, predominantly in L1-3. They do not co-express PV, SOM, or Cb, but half express CR (80%), a quarter reelin (VIP not tested). They display irregular or adapting firing patterns, exhibit a pronounced “sag” and innervate small dendritic shafts of both interneurones and pyramids (Human temporal cortex; Varga et al., 2015). Two classes of mouse L2/3 CR cells preferentially innervate interneurones: burst-firing, bipolar VIP/CR-cells and adapting, accommodating multipolar CR-cells and may be cortical equivalents of ISI-I and III respectively (Caputi et al., 2009).

**Projection neurones**: A small population (6–9%) of low threshold spiking SOM cells that also express NPY, nNOS and SPR form a distinct morphological class with long distance corticocortical or corticofugal projections (Yavorska and Wehr, 2016).

## Conclusion

The similarities between hippocampal CA regions and neocortical layers are striking: their development, the classes of neurones which result and the unidirectional flow of excitation through the regions and layers, which preserves the integrity of original signals. The prominent differences may result from a need for a far larger number of often smaller and simpler principal neurones in neocortex to perform a wider range of sophisticated computations, while avoiding the inefficiency of long, myelinated “local circuit” connections. Stacking principal cells in columns maximizes efficiency. However, this new arrangement presents new challenges, both to axons and dendrites that must make appropriate connections, to interneurones that must infiltrate this apparent chaos and to neuroscientists trying to understand the circuitry. Within these columns, myriad neuronal compartments, belonging to many neuronal classes, lie side by side. How do the axons that ramify there, or those simply passing through, choose from amongst these targets and how do postsynaptic compartments know which to accept? Understanding the mechanisms already apparent in simpler cortices, but hitherto largely unexplained; mechanisms that ensure the rejection of inappropriate and the formation of appropriate connections, each with its own unique signature, is an exciting challenge for the future.

## Author contributions

AM and AT wrote the manuscript and designed the figures.

### Conflict of interest statement

The authors declare that the research was conducted in the absence of any commercial or financial relationships that could be construed as a potential conflict of interest.
