# Supplementary material for: Cornu Ammonis Regions–Antecedents of Cortical Layers?
Source: Front Neuroanat. 2017 Sep 26;11:83. doi: 10.3389/fnana.2017.00083 (PMC5622992; doi:10.3389/fnana.2017.00083)
Supplement: Supplementary file 1 [file DataSheet1.pdf]

Supplementary figure 1

Neurolucida (Microbrightfield) 3D reconstructions of CA1 neurones that have been filled with biocytin and processed with Avidin-HRP following intracellular recordings in 450-500µm thick slices of adult rat hippocampus (for methods see Pawelzik *et al.*, 1999; 2002). Due to slicing, only the axons within the depth of the slice were recovered, though the dendrites are intact.
All dendrites are in dark pink and axons in light pink. Layer boundaries have been added, but only line up accurately with the neurone at the start and end of each video. Some somata are not fully reconstructed in 3D.
3D Reconstructions by: Joanne Falck, Georgia Economides and Sigrun Lange. Recordings, dye-filling and original drawing tube-reconstructions were by members of the lab listed in acknowledgements; in particular, Dr Hannelore Pawelzik, who recorded and filled numerous, elusive and sparsely distributed interneurones and cell-pairs, but died before many could be reconstructed.
Videos by: Adam Phillips and Svenja Falk

3D reconstruction of a CA1 pyramidal cell with its soma in *stratum pyramidale*, its axon branching in *stratum oriens* and the alveus where it excites pyramidal basal dendrites and a range of interneurones. Its basal dendrites are in *stratum oriens* and apical oblique dendrites in *stratum radiatum* where they receive inputs from Scaffer collateral axons. The apical dendritic tuft forms in *stratum lacunosum moleculare*, where the perforation path terminates. Dendritic spines were not included in this reconstruction. [980513B]
http://uclsop.net/interneuron-reconstruction/ca1-pyramid

3D reconstruction of a CA1 axo-axonic cell, with its cell body in *stratum pyramidale*, dendrites spanning all layers and axon confined to 'deep' *stratum pyramidale* and adjacent *stratum oriens* where it inhibits pyramidal axon initial segments. [970911C]
http://uclsop.net/interneuron-reconstruction/axo-axonic

3D reconstruction of a CA1 back projection cell, with its soma in and its horizontally oriented dendrites mostly confined to *stratum oriens* where it receives strong input from local pyramidal cells. Its local axon forms four terminatal arbours in: 1) CA1/CA2 *straum oriens*, 2) CA1 *stratum pyramidal,* 3) proximal CA2 *stratum radiatum*, 4) more distal CA1 *stratum radiatum* and *stratum lacunosum moleculare*. Two longer axonal branches can be seen leaving CA1, traversing CA2 and CA3 *stratum radiatum* and entering CA3 *stratum pyramidale*. Since this cell was filled in a slice, its more diatal projections could not be identified. [980120A]
http://uclsop.net/interneuron-reconstruction/backprojection

3D reconstruction of a CA1 wide arbour basket cell with its soma in *stratum pyramidale*, dendrites spanning all layers and axon confined to *stratum pyramidale* and immediately adjacent *stratum oriens* and *radiatum* where it inhibits pyramidal somata and very proximal dendrites. [031031 AM1]
http://uclsop.net/interneuron-reconstruction/basket

3D reconstruction of a CA1 *stratum oriens* bistratified cell with its soma and horizontal dendrites in *stratum oriens* and its axon ramifying in *stratum radiatum* and *oriens* where it inhibits pyramidal dendrites in the Schaffer collateral termination zone. [011023 HP2].
http://uclsop.net/interneuron-reconstruction/bistratified

3D reconstruction of a CA1 Ivy cell, with its soma in *stratum pyramidale,* its dendrites spanning proximal *stratum oriens* and *radiatum* and its dense axon ramifying predominantly in *stratum*

pyramidal and proximal *stratum oriens* and *radiatum* where it inhibits proximal, pyramidal dendrites. [970717D]
http://uclsop.net/interneuron-reconstruction/ivy

3D reconstruction of a CA1 OLM cell, with its soma and thorny dendrites in *stratum oriens* (where it receives excitatory input almost exclusively from CA1 pyramids) and its axons confined to *stratum lacunosum moleculare* where it inhibits distal pyramidal dendrites in the performant path termination zone. [011017 HP2]
http://uclsop.net/interneuron-reconstruction/olm

3D reconstruction of a CA1 perforant path associated cell with its soma close to the *stratum radiatum/lacunosum moleculare* border. Its dendrites and axon are largely confined to *stratum lacunosum moleculare* where they receive input from the perforant path and inhibit distal pyramidal dendrites in the perforant path termination zone. [011127 HP1]
http://uclsop.net/interneuron-reconstruction/ppa

3D reconstruction of a CA1 Schaffer collateral associated cell with its soma close to the *stratum radiatum/lacunosum moleculare* border, its sparse dendrites spanning all layers and its axon ramifying sparsely, mainly in *stratum radiatum and oriens* where (like bistratified cell axons) it inhibits intermediate pyramidal dendrites in the Schaffer collateral termination zone. [990611 HP2]
http://uclsop.net/interneuron-reconstruction/sca
